# The College of Nursing and the Nurses' Curriculum

**Published:** 1920-02-28

**Authors:** 


### THE MATRONS' AND SISTERS' DEPARTMENT.

### THE COLLEGE OF NURSING AND THE NURSES' CURRICULUM.

It must be confessed that the business of setting the probationer's course of studies has often been rather a happy-go-lucky one in the past. Hitherto there has been no standard. If the hospital authorities chose to consider physiology and anatomy useless for nursing purposes they could so arrange that only a modicum of these sciences ever descended to the level of the students. In some hospitals "materia medica" held an important place. In others it was conspicuous by its absence. No one was prepared to affirm whether invalid cookery were a necessity or a luxury in the nurse's course. In fact the leading factors in deciding whether a subject should be introduced into the curriculum used to be, first, the expense incurred, and, secondly, the wishes of the candidates themselves. If by amending the course more probationers could be inspired with the ambition of entering for it, well and good.

Under this lack of system the results were better than might be supposed. For matrons trained in the institutions which deservedly lead the way gradually carried the good seed of a well-thought-out training curriculum throughout the length and breadth of the land. Every reform achieved, every really well-organised training-centre owes its existence to the energies of a matron prepared for the good work by long residence in one of these hospitals or Poor-law infirmaries, where the business of training nurses has been brought to something bordering on perfection. The variety which has resulted from long experimentation in different directions has been all for the good. But the time has come for standardisation, and in many directions the authorities are turning to the College of Nursing for a definite pronouncement on vexed questions.

This week we are able to record that the authorities of the Royal United Hospital, Bath, have expressed their appreciation of the principles of training laid down by the College, and have in some directions modified their training in order to come into line. This process is going on all over the kingdom. The value of having a consultative body, able and willing to advise on matters relating to the training of nurses, is beginning to be understood. For hitherto there has been no means of getting expert advice in this difficult domain. It is true that the heads of successful training-schools have always done an immense deal of valuable work in this direction. They were liable to be appealed to not only by their own sisters when appointed to matronships, but also by public bodies, and the managers of institutions far removed from their own. Miss Luckes long acted as adviser to a quite unusual extent. But the pressure of work in their own department hinders matrons from helping their less experienced comrades as much as they would like. And there is inevitably a great deal of waste under this system. The careful calculations and adjustments of subjects to the time available for study needed in framing the course can be to some extent standardised and made available for all. It is not necessary to think these things out from the beginning every time. There is a great body now of received opinion in matters relating to the training of nurses, and of this the College of Nursing is the natural depositary, and the natural exponent. Their work focuses and radiates in countless directions the faithful experience of a whole generation of able matrons, experts in things concerning the training-school.

The noisy but numerically insignificant group which opposes the College would fain persuade nurses that registration pure and simple will accomplish for the nursing profession what other and wiser heads perceive must be the work of an inner professional organisation. The Registration Board can register results, but the processes through which those results are attained need the most careful study and continual readjustment. It must never be forgotten that nursing is no stationary profession. It follows hard on medical and surgical science, and must continually adapt itself to the advances of the senior services. Nothing can guide the smaller training-centres so surely as a council of men and women breathing the spirit of those institutions where the most recent discoveries are born and tried before they become public property. It is because the College of Nursing can command this concentrated inner view of modern progress in health matters that the judgment of the College on the things necessary for the training of good nurses is priceless. The surgeons and physicians who are hot on the scent of disease and are tracking it to its lairs must have nurses able to follow out their ideas. That is the crux of good training.

